# Linking preoperative and early intensive care unit data for prolonged intubation prediction

**DOI:** 10.3389/fcvm.2024.1342586

**Published:** 2024-03-26

**Authors:** Yuqiang Wang, Shihui Zhu, Xiaoli Liu, Bochao Zhao, Xiu Zhang, Zeruxin Luo, Peizhao Liu, Yingqiang Guo, Zhengbo Zhang, Pengming Yu

**Affiliations:** ^1^ Cardiovascular Surgery Research Laboratory, West China Hospital, Sichuan University, Chengdu, China; ^2^ Mailman School of Public Health, Columbia University, New York, NY, United States; ^3^ Center for Artificial Intelligence in Medicine, The General Hospital of PLA, Beijing, China; ^4^ School of Automation, University of Science and Technology Beijing, Beijing, China; ^5^ Rehabilitation Medicine Center, West China Hospital, Sichuan University, Chengdu, China; ^6^ Research Institute of General Surgery, Jinling Hospital, Medical School of Nanjing University, Nanjing, China

**Keywords:** prolonged intubation, cardiac surgery, multicenter, nomogram, intensive care unit

## Abstract

**Objectives:**

Prolonged intubation (PI) is a frequently encountered severe complication among patients following cardiac surgery (CS). Solely concentrating on preoperative data, devoid of sufficient consideration for the ongoing impact of surgical, anesthetic, and cardiopulmonary bypass procedures on subsequent respiratory system function, could potentially compromise the predictive accuracy of disease prognosis. In response to this challenge, we formulated and externally validated an intelligible prediction model tailored for CS patients, leveraging both preoperative information and early intensive care unit (ICU) data to facilitate early prophylaxis for PI.

**Methods:**

We conducted a retrospective cohort study, analyzing adult patients who underwent CS and utilizing data from two publicly available ICU databases, namely, the Medical Information Mart for Intensive Care and the eICU Collaborative Research Database. PI was defined as necessitating intubation for over 24 h. The predictive model was constructed using multivariable logistic regression. External validation of the model's predictive performance was conducted, and the findings were elucidated through visualization techniques.

**Results:**

The incidence rates of PI in the training, testing, and external validation cohorts were 11.8%, 12.1%, and 17.5%, respectively. We identified 11 predictive factors associated with PI following CS: plateau pressure [odds ratio (OR), 1.133; 95% confidence interval (CI), 1.111–1.157], lactate level (OR, 1.131; 95% CI, 1.067–1.2), Charlson Comorbidity Index (OR, 1.166; 95% CI, 1.115–1.219), Sequential Organ Failure Assessment score (OR, 1.096; 95% CI, 1.061–1.132), central venous pressure (OR, 1.052; 95% CI, 1.033–1.073), anion gap (OR, 1.075; 95% CI, 1.043–1.107), positive end-expiratory pressure (OR, 1.087; 95% CI, 1.047–1.129), vasopressor usage (OR, 1.521; 95% CI, 1.23–1.879), Visual Analog Scale score (OR, 0.928; 95% CI, 0.893–0.964), pH value (OR, 0.757; 95% CI, 0.629–0.913), and blood urea nitrogen level (OR, 1.011; 95% CI, 1.003–1.02). The model exhibited an area under the receiver operating characteristic curve (AUROC) of 0.853 (95% CI, 0.840–0.865) in the training cohort, 0.867 (95% CI, 0.853–0.882) in the testing cohort, and 0.704 (95% CI, 0.679–0.727) in the external validation cohort.

**Conclusions:**

Through multicenter internal and external validation, our model, which integrates early ICU data and preoperative information, exhibited outstanding discriminative capability. This integration allows for the accurate assessment of PI risk in the initial phases following CS, facilitating timely interventions to mitigate adverse outcomes.

## 1 Introduction

For cardiac surgery (CS), the sternum-median incision represents an invasive procedure. Following surgery, cardiac patients are typically transferred to the intensive care unit (ICU) and placed on invasive mechanical ventilation (MV) (1). The Society of Thoracic Surgeons (STS) defines prolonged intubation (PI) as requiring intubation for more than 24 h (2). PI can lead to extended ICU stays, an increased 30-day mortality rate for CS patients (14% vs. 1%, *p* < 0.05), and heightened rates of ineffective medical interventions (3). Consequently, the development of a reliable postoperative PI prediction model and the implementation of targeted early interventions for high-risk patients would be immensely advantageous.

The STS has recently utilized preoperative data to construct a scoring system for postoperative outcomes in CS, including PI (2). However, postoperative complications encompass multiple phases. Many existing models primarily focus on predicting outcomes based solely on preoperative data, thus limiting their applicability in clinical settings (4). Postoperative outcomes are influenced not only by the patient's preoperative medical condition but also by the potential risks introduced by the surgical procedure itself and the management of anesthesia (5). It is important to note the significant role of the perfusionist in cardiac surgical procedures (6). Failure to adequately account for the persistent impact of surgical, anesthetic, and cardiopulmonary bypass procedures on subsequent respiratory function can compromise the performance of disease prediction models (7). Recent findings by Brescia indicate that much of the variation in pneumonia incidence remains unexplained by predictive models focused solely on the type of CS and preoperative risk factors. These results suggest that other unmeasured factors related to medical practices may contribute to the observed variation (8). We hypothesize that the intraoperative process involves complex factors that are challenging to fully capture while standardizing data presents its own set of difficulties. Notably, the ongoing disruption of the respiratory system by surgery, anesthesia, cardiopulmonary bypass, and human factors is ultimately reflected in early physiological indicators (such as urine output, blood gases, and tissue perfusion) and aspects of ICU management (e.g., hemodynamics, vasopressor usage, and ventilator settings) following ICU admission (9).

The objective of this study was to integrate early ICU data (collected within 12 h of ICU admission) with preoperative data to develop and externally validate an explanatory and generalizable predictive model for PI in CS patients. The goal was to assess the risk of early respiratory system deterioration in CS patients and to offer a valuable 12-h intervention window in the initial stages for intensivists.

## 2 Methods

### 2.1 Study designs, cohorts, and outcomes

In this retrospective study, we focused on patients admitted to the ICU following coronary artery bypass grafting (CABG), valve surgery, or a combination of these procedures. We defined PI as requiring intubation for more than 24 h in accordance with the latest guidelines from the STS (2). The study design included the development of an explainable prediction model, its external validation, and the assessment of its predictive performance using multiple metrics.

**Figure 1:**
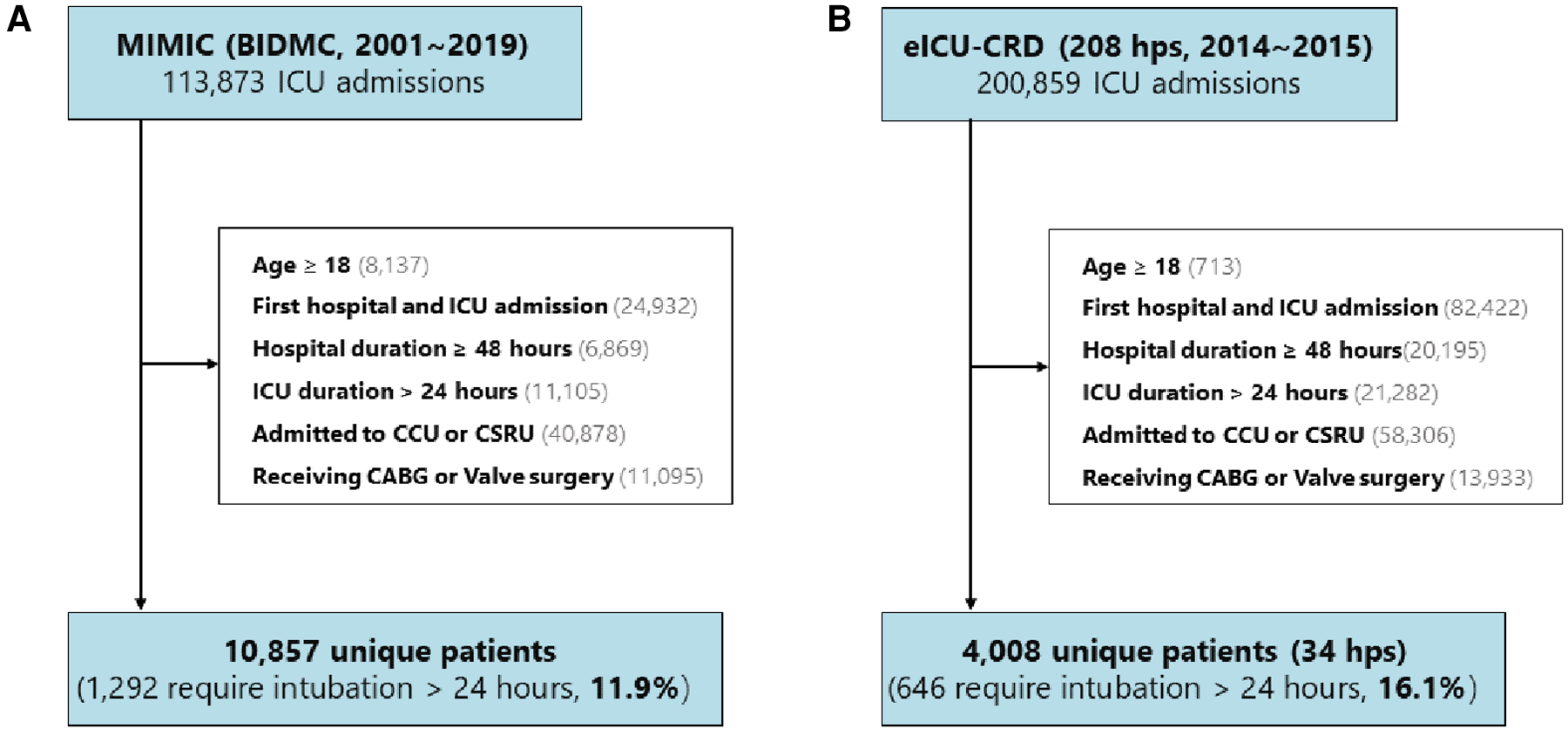
Study design and cohorts. The study encompassed (**A**) 10,857 (11.9%) patients from the MIMIC database and (**B**) 4,008 (16.1%) patients from 34 hospitals included in the eICU-CRD. MIMIC, Medical Information Mart for Intensive Care; BIDMIC, Beth Israel Deaconess Medical Center; eICU-CRD, eICU Collaborative Research Database; hps, hospital; CCU, coronary care unit; CSRU, Cardiac Surgery Recovery Unit; CABG, coronary artery bypass grafting.

We utilized two publicly available ICU databases for our analysis: the Medical Information Mart for Intensive Care (MIMIC), which encompasses MIMIC-III CareVue and MIMIC-IV (2001–2019), and the eICU Collaborative Research Database (eICU-CRD, 2014–2015). The selection criteria for both databases are illustrated in Figures 1A,B. The eligibility criteria required patients to be over 18 years of age, to have their first hospital and ICU admissions, to have a hospital stay exceeding 48 h, to have an ICU stay exceeding 24 h, to have undergone CABG or valve surgery, and to have been admitted to the cardiac care unit (CCU) or cardiac surgery recovery unit (CSRU) postsurgery. Consequently, 10,857 (11.9%) patients from MIMIC and 4,008 (16.1%) patients from 34 hospitals in the eICU-CRD were included in the study.

To construct the development set, we randomly selected half of the patients from MIMIC and patients from six hospitals, considering the number of patients, hospital type (teaching or non-teaching), and region in the eICU-CRD. The hospital IDs were used to train the model, including 122, 176, 382, 413, 416, and 420. A detailed description is presented in Supplementary Table S1. Subsequently, the development set was divided into a training set (80%) and a test set (20%). The remaining patients from the additional 28 hospitals in the eICU-CRD were allocated to the external validation datasets. The primary outcome of interest was the occurrence of PI.

### 2.2 Feature construction and imputation

Patient information was collected within the initial 12-h period, encompassing baseline data, administered treatments, laboratory results, vital signs, and clinical scores, as detailed in the supplementary table (Supplementary Table S2). The missing ratio for each variable was computed and is documented in Supplementary Table S3. Variables with more than 50% missing values were excluded from subsequent analyses.

Basic clinical and demographic information included age, admission type, BMI, ethnicity, height, weight, gender, and pre-ICU admission days. Categorical variables, such as CABG surgery and valve surgery, along with vasopressor usage, were employed to denote the treatment received. Admission type was categorized based on whether the patient was an unplanned admission or not. Continuous variables associated with mechanical ventilation, including plateau pressure, tidal volume, and positive end-expiratory pressure (PEEP), were also integrated into the analysis. For specific laboratory parameters—such as albumin, base excess, bicarbonate, chloride, hemoglobin, international normalized ratio (INR), neutrophil count, partial pressure of oxygen in the artery (PaO_2_), PaO_2_/fraction of inspired O_2_ratio (FiO_2_), pH level, and platelet count—minimum values were selected. Conversely, for parameters like anion gap, bilirubin, brain natriuretic peptide (BNP), blood urea nitrogen (BUN), creatinine, hematocrit, lactate, magnesium, partial pressure of carbon dioxide in the artery (PaCO_2_), potassium, prothrombin time (PT), partial thromboplastin time (PTT), sodium, troponin, white blood cell (WBC) count, PEEP, plateau pressure, and tidal volume, maximum values were utilized. Lymphocytes were represented by the mean values. The patient's vital signs, encompassing mean central venous pressure (CVP), heart rate, respiratory rate, systolic blood pressure (SBP), and temperature, along with minimum pulse oxygen saturation (SpO_2_), Glasgow Coma Scale (GCS), and maximum FiO_2_, were also considered in the analysis. This included the summation of urine output (UO), alongside clinical scores such as Charlson Comorbidity Index (CCI), peak pain status [evaluating pain levels using the Visual Analog Scale (VAS)], and Sequential Organ Failure Assessment (SOFA) score.

Missing data were imputed utilizing a random forest-based imputation model called Multiple Imputation by Chained Equations, implemented through the miceforest library in Python (version 3.7). The algorithm employs a lightGBM random forest, iteratively imputing missing values through chained equations across a specified number of datasets within the entire training set. The imputed data were then used to calculate missing values for the PaO_2_/FiO_2_ ratio, BMI, and SpO_2_/FiO_2_ ratio.

### 2.3 Important feature selection

Feature selection was conducted through a combination of univariate analysis and LASSO logistic regression (LR), employing 10-fold cross-validation (CV). The variance inflation factor (VIF) was also utilized to identify multicollinearity. The performance of the CV was evaluated based on the area under the receiver operating characteristic (AUROC) curve.

### 2.4 Predictive model construction and evaluation

For the multivariable analysis, we employed binary logistic regression (LR) with 10-fold CV to assess the relationship between the selected variables and the outcome. Subsequently, the trained model was utilized for predictions on the training, test, and external validation sets. A forest plot was employed to visualize the odds ratio (OR). Various evaluation metrics, including the receiver operating characteristic (ROC) curve, the precision–recall (PR) curve, AUCROC, the area under the precision–recall curve (AUPRC), sensitivity, and specificity, were applied. Decision curve analysis (DCA) was used to evaluate the model's applicability across different datasets without additional information. ROC, PR, and calibration curves were generated to compare model performance across different ensembles. The prediction of PI was represented visually through a nomogram using the selected variables, with the inverse logistic regression implemented as the nomogram function.

### 2.5 Statistical analysis

Continuous variables and ordinal variables were represented as the median value and the interquartile range (IQR), while categorical variables were presented as numbers with corresponding percentages. Non-parametric tests were utilized for the difference test, employing the R package gtsummary version 1.4.2 (https://cloud.r-project.org/web/packages/gtsummary) in R software version 4.1.1. Specifically, the Kruskal–Wallis test was employed for comparing continuous variables across two or more samples, and the *χ*^2^ test was utilized for comparing categorical variables. For the calculated metrics, 95% confidence intervals (CIs) were computed through 2,000 stratified bootstrap replications using the Python scikit-learn package version 1.0.2 (https://scikit-learn.org/stable/) with Python software version 3.7.16. A significance level of *p* < 0.05 in a two-sided test was considered statistically significant.

## 3 Results

### 3.1 Study cohorts and characteristics

The study cohorts comprised 6,498 PI patients from the training set (event rate 11.8%), 5,429 PI patients from the test set (event rate 12.1%), and 2,938 PI patients from the external validation set (event rate 17.5%). Table 1 presents the characteristics of these cohorts across different groups. The performance stratified by outcomes is detailed for all study cohorts in Supplementary Tables S4–S6. Patients with PI exhibited distinctive features compared to non-PI patients, showing tendencies to be older, possess higher BMI, undergo valve surgery or mixed surgeries, and have elevated levels of PEEP, plateau pressure, tidal volume setting, and CCI score.

**Table 1:** Characteristics of three study cohorts.

Variable	Training set (6,498, 11.8%)	Test set (5,429, 12.1%)	External validation set (2,938, 17.5%)
Age (years)	68.0 (60.0, 76.0)	68.0 (60.0, 76.0)	68.0 (60.0, 77.0)
Female, *n* (%)	1,935 (29.8)	1,583 (29.2)	1,005 (34.2)
BMI (kg/m^2^)	28.6 (25.3, 32.6)	28.7 (25.5, 32.6)	28.9 (25.2, 33.6)
Unplanned admission, *n* (%)	4,651 (71.6)	3,874 (71.4)	2,258 (76.9)
Ethnicity, *n* (%)			
Asian	147 (2.3)	114 (2.1)	37 (1.3)
Black	234 (3.6)	182 (3.4)	246 (8.4)
Hispanic	148 (2.3)	133 (2.4)	103 (3.5)
Other	1,174 (18.1)	1,134 (20.9)	164 (5.6)
White	4,795 (73.8)	3,866 (71.2)	2,388 (81.3)
CCI score	4.0 (3.0, 6.0)	5.0 (4.0, 6.0)	3.0 (2.0, 4.0)
SOFA score	6.0 (4.0, 8.0)	6.0 (4.0, 8.0)	7.0 (5.0, 9.0)
CABG, *n* (%)	4,588 (70.6)	3,901 (71.9)	1,866 (63.5)
Valve surgery, *n* (%)	2,928 (45.1)	2,334 (43.0)	1,432 (48.7)
CABG and valve, *n* (%)	1,018 (15.7)	806 (14.8)	360 (12.3)
Vasopressor, *n* (%)	1,609 (24.8)	1,498 (27.6)	413 (14.1)
PEEP (cmH_2_O)	5.0 (5.0, 5.0)	5.0 (5.0, 5.0)	5.0 (5.0, 5.0)
Plateau pressure (cmH_2_O)	18.0 (14.0, 22.0)	18.0 (13.0, 22.0)	20.0 (17.0, 23.0)
Tidal volume (mL)	553.0 (443.0, 658.0)	550.0 (406.0, 652.0)	580.0 (500.0, 664.8)
Invasive MV (h)	6.4 (2.3, 15.1)	6.2 (2.0, 15.3)	7.8 (3.6, 17.6)
Invasive MV > 48 h, *n* (%)	390 (6.0)	370 (6.8)	246 (8.4)
VAS	4.0 (2.0, 6.0)	5.0 (3.0, 7.0)	4.0 (1.0, 5.0)
Pre ICU admission (days)	0.7 (0.2, 2.7)	0.8 (0.1, 2.8)	0.4 (0.3, 2.2)
ICU duration (days)	2.1 (1.3, 3.3)	2.1 (1.3, 3.4)	2.1 (1.4, 3.4)
Hospital duration (days)	7.2 (5.3, 10.5)	7.3 (5.4, 10.8)	7.2 (5.3, 10.5)

CCI, Charlson Comorbidity Index; SOFA, Sequential Organ Failure Assessment; ICU, intensive care unit; CABG, coronary artery bypass grafting; PEEP, positive end-expiratory pressure; MV, mechanical ventilation; VAS, Visual Analog Scale.

### 3.2 Feature selection and model building

**Figure 2:**
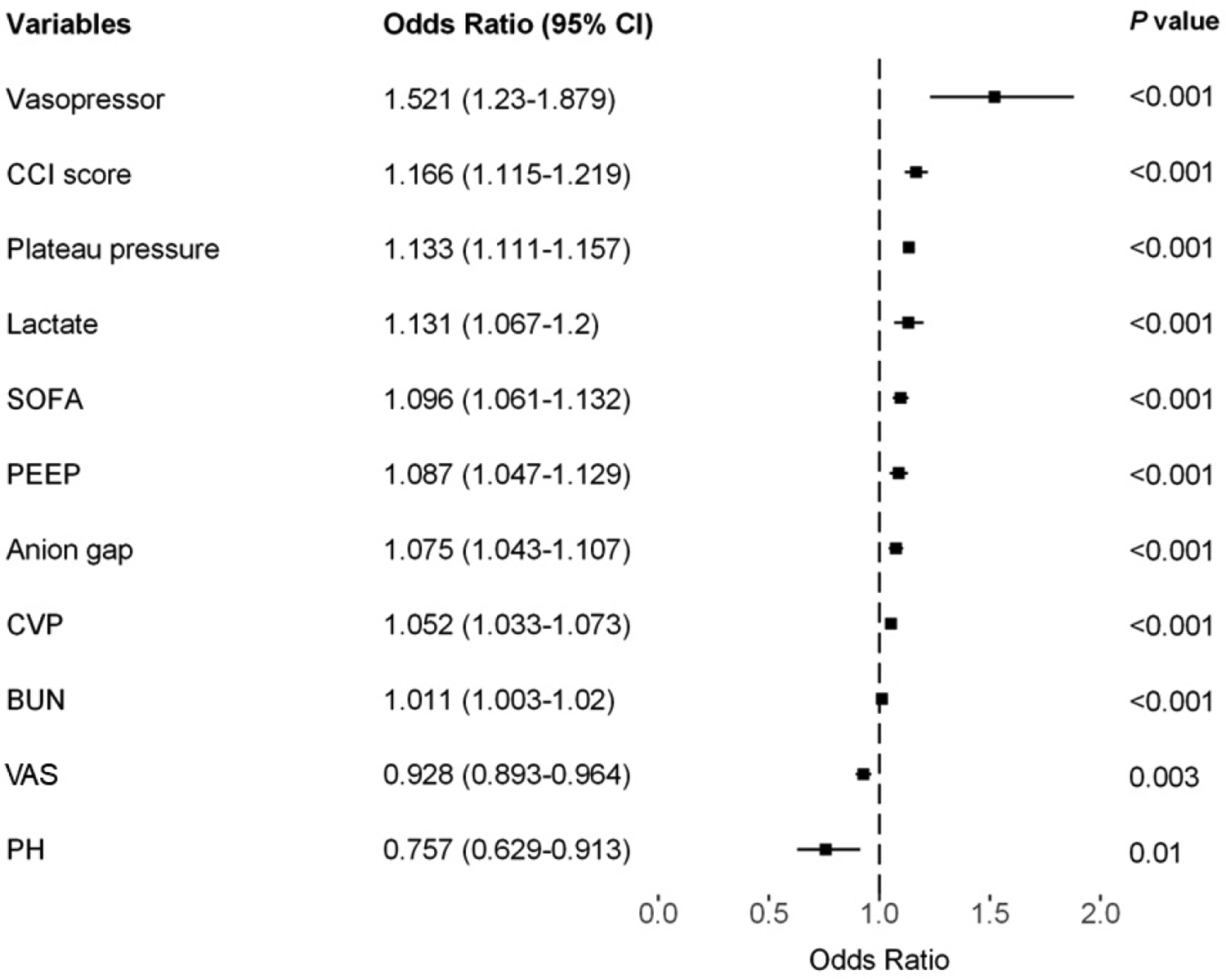
Forest plot of multivariable analysis for risk factors of PI.

**Figure 3:**
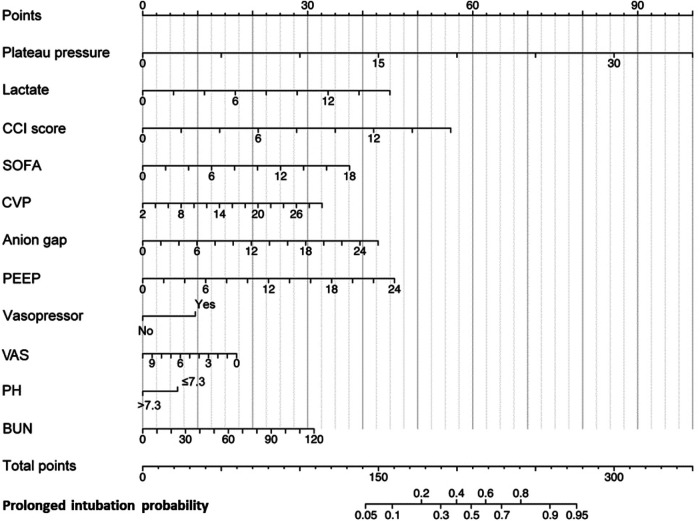
Nomogram for early predicting probability of PI. CCI, Charlson Comorbidity Index; SOFA, Sequential Organ Failure Assessment; CVP, central venous pressure; PEEP, positive end-expiratory pressure; VAS, Visual Analog Scale; BUN, blood urea nitrogen.

The LASSO LR implemented coefficient shrinkage property, resulting in a more stable set of selected variables. The one standard error rule was employed to select the lambda that produced the most parsimonious model with comparable performance to the best model (Supplementary Figures S1A,B). Out of the 39 variables with non-zero coefficients, 12 were selected. Subsequently, through the application of univariable regression, 11 variables—excluding UO (OR = 1)—were all found to be significantly significant (Supplementary Figure S2). Furthermore, the VIF for all considered variables was less than 5 (Supplementary Table S7). For the prediction of PI, the final model was the LR model comprising 11 variables, trained using the training set. Odds ratios from the multivariate analysis are displayed in Figure 2. This finding was largely consistent with the univariate analysis results. Vasopressor usage and increases in the CCI score, plateau pressure, lactate, SOFA score, PEEP, anion gap, CVP, and BUN were associated with greater risks of developing PI. In contrast, lower pH levels or VAS scores was associated with higher PI risks. A nomogram generated using the R package rms version 6.5.0 (https://cran.r-project.org/web/packages/rms) is depicted in Figure 3. The nomogram was utilized to facilitate manual PI prediction for each patient and provide enhanced visualization of the effect of coefficients in the LR model. Given the values of the 11 variables of an individual patient, one can obtain a prediction score by matching each value to its corresponding scale in the nomogram. The rms package utilizes the coefficients of the trained LR model to compute the point scale of the nomogram.

### 3.3 Predictive performance of discrimination and calibration

**Figure 4:**
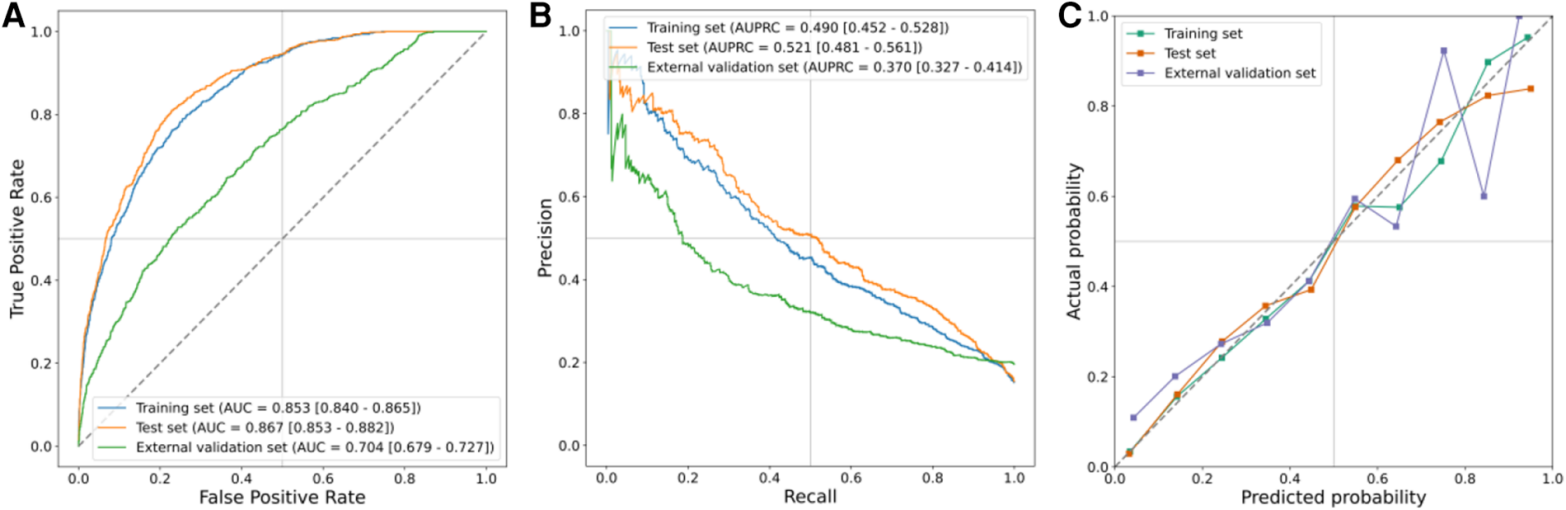
Prediction performance in training, test, and external validation sets. (**A**) AUROCs, (**B**) AUPRCs, and (**C**) calibration curves.

The results of the LR model on the training, test, and external validation sets are presented in Figures 4A,B, while the corresponding 95% CIs are listed in Supplementary Table S8. Notably, the LR model exhibited optimal performance on the test set. These metrics collectively indicated that the model performed effectively across all ensembles. The AUROC was 0.853 (95% CI, 0.840–0.865) in the training set, 0.867 (95% CI, 0.853–0.882) in the test set, and 0.704 (95% CI, 0.6791–0.727) in the external validation set. Similarly, the area under the AUPRC was 0.490 (95% CI, 0.452–0.528) in the training set, 0.521 (95% CI, 0.481–0.561) in the test set, and 0.370 (95% CI, 0.327–0.414) in the external validation set. Figure 4C illustrates the calibration curve of the LR model, showcasing particularly robust calibration on the training and test sets. However, for external validation sets, the curves exhibited deviation at the higher end when higher probabilities were predicted.

In Supplementary Figure S3, the DCA outputs indicated the applicability of our model to other datasets with varying occurrence rates. The DCAs consistently demonstrated net benefits, surpassing both the treat-all and treat-none strategies. The prediction model exhibited comparability to the treat-all at low cutoff probabilities and resembled the treat-none at high cutoff probabilities. Notably, the curve of the external validation set displayed a slightly lower net benefit compared to the treat-all curve when the threshold probability was <10%.

## 4 Discussion

**Figure 5:**
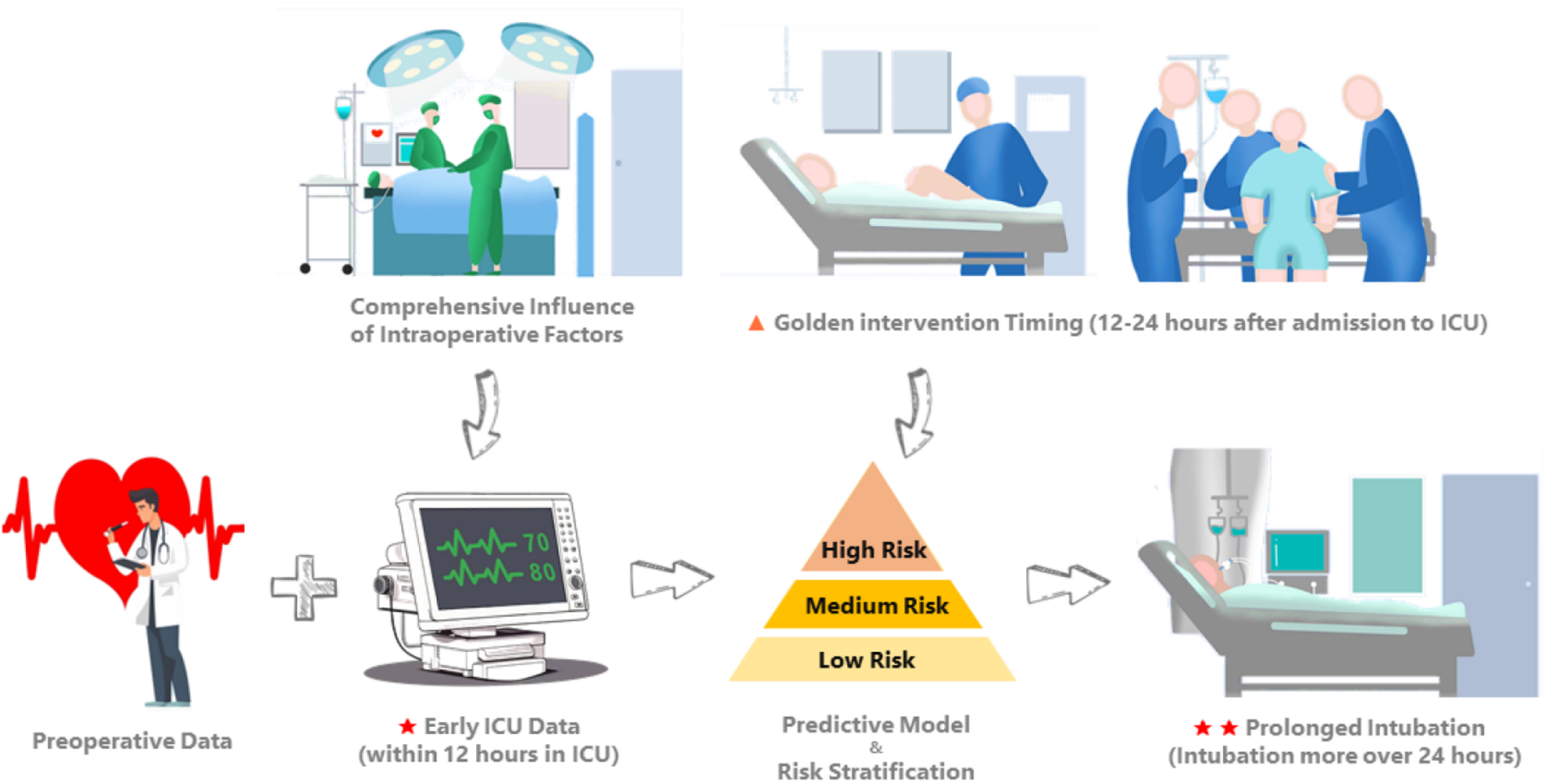
Comprehensive influence of intraoperative factors is ultimately reflected in the early physiologic measures and management strategies after the patient is admitted to the ICU. Incorporating early ICU data (within 12 h of ICU admission) with preoperative data, we developed and externally validated an interpretable predictive model for PI in CS patients requiring intubation for more than 24 h. Our objective was to promptly identify the risk of early respiratory system deterioration in CS patients and delineate an optimal intervention window (12–24 h after ICU admission) for intensivists.

This study aimed to develop and validate a predictive model for PI, defined as requiring intubation for more than 24 h following CS. The model was based on merged multicenter datasets from the MIMIC and eICU-CRD. Previous studies have primarily focused on identifying risk factors for PI, predominantly analyzing preoperative and intraoperative variables (4, 10). However, they rarely incorporated early postoperative physiological data, such as measurements taken shortly after ICU admission. Moreover, limitations in the cohort size and external validation have constrained the performance of existing models. To address these gaps, we developed the first predictive model for PI after CS utilizing early postoperative physiological data. Our study specifically focused on utilizing readily available physiological variables measured within 12 h of ICU admission to predict PI, aiming to elucidate early respiratory system deterioration in patients. Our objective was to establish a model based on routinely collected data and provide a predictive tool that is both effective and interpretable for surgical teams. This tool will facilitate the early identification of high-risk patients requiring PI, enabling timely intervention strategies (Figure 5).

In the context of “Fast-Track cardiac surgery,” early extubation within 6 h postoperatively serves as a benchmark (11). However, some patients may experience PI due to the disease itself, surgical procedures, and perioperative management. Due to variations in the definition of PI (ranging from 24 h to 14 days), previous studies have reported incidence rates of PI after CS ranging from 2.6% to 22.7% (3). This prolongs patient hospitalization and increases mortality rates while also delaying long-term functional recovery, thereby imposing a significant medical burden on patients. For several reasons, we opted for a 24-h threshold to define PI. The latest guidelines from the STS consider intubation duration exceeding 24 h as a major endpoint following CS (2). In addition, this threshold helps us address challenges such as slow sedation recovery, weak maintenance of internal environmental stability, insufficient human resources, and adherence to extubation policies and protocols across different medical centers, all of which could contribute to PI time. Furthermore, we posit that the study population primarily comprises patients undergoing elective CS, whose conditions are relatively stable compared to emergency cases. Thus, setting excessively long thresholds (e.g., 48 h or longer) to define PI may lead to underestimating this complication.

Facing the high complexity and collinearity of physiological data, machine learning (ML) methods can capture the non-linear relationship between complex data and construct predictive models (12–14). However, challenges persist. On one hand, predictive models are probabilistic and may inadvertently select or deselect “risk factors” (15). Each predictive model requires internal and external validation with patient cohorts to assess internal stability and external generalizability (16). Limited multicenter data studies for external validation exist, constraining confidence in the effectiveness and generalizability of predictive models (17). On the other hand, tools for the interpretability of predictive models, such as feature importance ranking, explanation of model inference, or interpretation of model results, are lacking (18, 19). This hampers the translation of predictive models into clinical use, undoubtedly widening the gap in their integration into real clinical practice (17). This disparity is evident in the significant difference between the number of published prediction models in 2019 (*n* = 12,422) and the number of algorithms approved for clinical use by the FDA in 2020 (*n* = 130) (17). The STS risk model uses clinically standardized data to characterize preoperative patient comorbidities for predictive purposes (2). Different timing of the model's predictions provides varying risk stratification at each stage. Most models are primarily designed to predict on a single time point (preoperative), limiting their application in the clinical setting (4). Cai et al. found that prediction models incorporating both preoperative and postoperative predictors demonstrated higher prediction accuracy than models relying solely on preoperative characteristics (20). Models based on both pre- and postoperative variables may offer dynamic risk stratification to guide flexible prophylaxis. To date, no study has predicted PI after CS by analyzing early physiological data transferred to the ICU.

We utilized a cohort of 6,498 patients from seven hospitals in our study to develop a model for explanatory prediction of PI based on preoperative status and records within the first 12 h of ICU admission. The model underwent evaluation through an internal validation set (5,429 patients) and an external validation set using a separate cohort (2,938 patients, 28 hospitals). In our study, the incidence rates of PI in the training, internal validation, and external validation cohorts were 11.8%, 12.1%, and 17.5%, respectively, consistent with other reports (10). Across all study cohorts, the discriminatory ability and clinical decision-making advantages of the model were consistently favorable (Figure 4 and Supplementary Figure S3). Disease prediction models exhibit good discriminative ability in predicting PI after CS. The AUROCs were 0.853 (95% CI, 0.840–0.865) in the training set, 0.867 (95% CI, 0.853–0.882) in the test set, and 0.704 (95% CI, 0.679–0.727) in the external validation set. Furthermore, multivariable analysis and a nomogram were employed to enhance the interpretability and intuitive understanding of the model. Eleven important risk factors were identified to early identify the high risk of PI, including plateau pressure, lactate, CCI score, SOFA, CVP, anion gap, PEEP, VAS, vasopressor usage, pH, and BUN. Early prediction of PI identifies high-risk patients entering the ICU and helps clinicians appropriately allocate intensive care resources.

Several studies have documented severe cases in which patients experience unstable hemodynamics, necessitating the administration of positive inotropic agents or vasopressors and prolonged mechanical ventilation via tracheal intubation (21). Elevated CVP has been shown to correlate with mortality, length of stay, and duration of MV in a large heterogeneous cohort of patients admitted to the ICU (22). The majority of patients experiencing severe respiratory failure exhibit varying degrees of inadequate tissue perfusion and impaired oxygenation, ultimately resulting in increased lactate production or anion gap, which are considered independent risk factors for hospitalization and all-cause mortality in critically ill patients with cardiac disease (23, 24).

Plateau pressure serves as a common metric for assessing lung compliance. Maintaining low plateau pressures is crucial for lung-protective ventilation, as elevated plateau pressures may precipitate ventilator-induced lung injury (25). In extensive surgical investigations, Thomas Bluth and his collaborators observed that although employing a high PEEP strategy consistently enhanced intraoperative oxygenation and respiratory maneuvers, it did not yield improvements in outcomes pertaining to postoperative pulmonary complications (26). Consequently, a recent consensus among experts suggests that intraoperative PEEP should be capped at a low fixed level as a standard intraoperative practice (27).

For decades, MV has been pivotal in clinical research and practice. However, reports indicate that the 1-year postdischarge survival rate for patients undergoing long-term MV is below 50% (28). Assessing the risk of PI in patients is of interest to clinicians and patients alike. While previous studies have identified numerous risk factors (4, 10), it is important to note that CS encompasses varied surgical techniques, cardiopulmonary bypass methods, and surgical access types, each exerting distinct effects on postoperative outcomes and clinical trajectories, potentially prolonging the intubation duration (29, 30). Furthermore, advancements in modern cardiac anesthesia practices, such as optimized intraoperative temperature management, fast-track anesthesia, and ventilator protocols (31), indisputably impact the postoperative intubation duration (9). Human factors also play a crucial role (32, 33). For instance, patients with cardiac valve disease devoid of underlying conditions and with satisfactory lung function are theoretically at low risk for PI. However, cases involving significant intraoperative bleeding or repeated aortic clamping necessitate extended ventilatory support upon return to the ICU after surgery, introducing bias in predictive modeling. The cumulative effect of various factors on patient physiology eventually influences early ICU interventions (34). In essence, intraoperative procedures may directly or indirectly contribute to the prolonged postoperative intubation duration (35, 36). Regrettably, existing predictive models for postoperative cardiac PI yield suboptimal results. Few researchers have examined the cumulative comprehensive impact of intraoperative adverse factors on postoperative patient outcomes. For instance, Atlas et al. assembled 918 patients from a single center to predict pulmonary complications occurring 24–72 h after elective cardiac surgery, achieving an AUROC of 0.70–0.75 (37). Building upon this, Sharma et al. employed a significantly larger sample size of cardiac surgical patients (32,045) to develop prediction models for prolonged ventilation following CS, achieving an AUROC of 0.787 (38). This may be attributed to limitations in the sample size and the intricate physiological changes during prolonged surgery under cardiopulmonary bypass (35).

Hence, we developed a PI prediction model by incorporating early ICU data with the patient's preoperative condition. This approach allowed us to circumvent the challenges associated with intraoperative data collection, which often yields sparse effects and requires intricate normalization. Instead, we acknowledged that early ICU data could indirectly capture the cumulative impact of intraoperative interventions. Our study facilitates a more intuitive assessment of PI risk for patients upon ICU admission. High risk denotes a heightened likelihood of requiring a prolonged postoperative intubation duration, underscoring the importance of diligently optimizing ventilation parameters and preparing for extubation. Conversely, low risk suggests the potential for swift extubation and liberation from invasive ventilation, emphasizing the need for vigilance regarding potential postoperative complications that could impede extubation (39).

Guidelines advocating for the early liberation of patients from invasive mechanical ventilation recommend implementing protocols such as spontaneous breathing trials, early mobilization, physiotherapy, and reduced sedation (40). PI following CS may predispose patients to complications such as dysphagia, aspiration pneumonia, and neurological issues associated with prolonged sedation (41). Previous studies have demonstrated that early multimodal rehabilitation intervention programs can reduce the duration of MV after CS from 22 h to 15 h (42). Early identification of high-risk patients and timely intervention are paramount. Therefore, the development of a reliable postoperative PI prediction model and the implementation of early targeted interventions for high-risk patients would be advantageous.

Invasive mechanical ventilation plays a crucial role as a supportive therapy for critically ill patients suffering from respiratory failure. These patients manifest diversity in disease etiology, pulmonary pathophysiology, and respiratory mechanics (43). Leveraging the widespread use of electronic monitoring and recording in high-income countries, along with the wealth of data generated during mechanical ventilation of ICU patients, artificial intelligence (AI) emerges as a promising avenue for optimizing mechanical ventilation modes. Previous studies have underscored the capacity of AI to forecast sepsis, circulatory failure, and mortality, highlighting its potential for further deployment in critical care settings. Notably, the development of more robust predictive models necessitates adherence to key conditions, listed as follows (17, 44): (a) external validation to ensure the reliability and generalizability of predictive models across different geographical regions; (b) calibration of predictive models to translate model outcomes into actionable insights at the patient level; and (c) enhancement of model interpretability to facilitate their bedside application. This interpretability aids physicians in the early identification of critical factors contributing to patient complications, including modifiable variables. Armed with such insights, clinicians can promptly discern potential risk factors for complications and devise evidence-based treatment strategies to mitigate them. The implications of our findings are manifold, bearing significant relevance for the clinical management of respiratory system complications in CS patients and guiding future research endeavors. Importantly, they have the potential to drive progress in the perioperative management of CS patients, encompassing improved preoperative optimization of high-risk individuals, prognostication of ICU workload and subsequent hospital expenditures, and the refinement of PI protocols tailored to high-risk patients.

Acknowledging that no individual predictive model can achieve flawless risk stratification is imperative. Dependence solely on predictors is unwarranted. Clinical expertise must be preserved, along with the capability to identify potentially life-threatening occurrences, to supplement these risk evaluation instruments and guarantee superior perioperative care. The significance of physiological measurements in the ICU and the selected ventilatory parameters cannot be overstated and warrants further investigation in subsequent studies. Furthermore, additional research on causal analysis is needed to further explore the important predictor variables found by the data-driven model, which could further facilitate the decision-supporting and early intervention for postoperative rehabilitation (45, 46).

### 4.1 Limitation

Our study encounters several limitations. First, it assumes a retrospective design, introducing potential selection bias in patient recruitment and data collection. The inclusion criteria focused solely on patients undergoing CABG or valve surgery, potentially affecting the applicability and accuracy of the model when extrapolating to other CS patients. Second, despite external validation in 28 U.S. hospitals, the validation cohort remains confined to Europe and Asia. Given that the success of CS hinges on comprehensive team support, the existing variations in decision-making and postoperative management across institutions exert significant influence. Third, the research on dynamic risk assessment models, such as the long short-term memory-based and transformer-based models, could potentially further improve the predictive performance of the model and the duration of early identification. Finally, our model exhibited poorer performance in external compared to internal validation, possibly attributed to differing proportions of missing data in the two cohorts. To support this conjecture, Gupta et al. developed a risk calculator for postoperative cardiac risk prediction based on 211,410 patients, noting a similar decline in AUROC when externally tested (47). This underscores the need for a thorough analysis of contributing factors and enhancement of the generalizability of our risk-scoring system.

## 5 Conclusion

Our predictive model offers both generalizability and precision, presenting a straightforward and visual tool for intensivists to estimate the risk of PI early in the ICU, enabling timely intervention within a critical 12-h window. This study serves as a proof of concept that postoperative infection following cardiac surgery can be accurately predicted by incorporating early ICU information with readily available preoperative data, thereby optimizing decision-making for patients undergoing CS with PI.

## Data Availability

The raw data supporting the conclusions of this article will be made available by the authors without undue reservation.
