# Supplementary material for: Linking preoperative and early intensive care unit data for prolonged intubation prediction
Source: Front Cardiovasc Med. 2024 Mar 26;11:1342586. doi: 10.3389/fcvm.2024.1342586 (PMC11005457; doi:10.3389/fcvm.2024.1342586)
Supplement: Supplementary file 1 [file Datasheet1.pdf]

Supplementary Material

1 Supplementary Figures and Tables

1.1 Supplementary Tables

Table S1 Hospital description of the selected cohort from eICU-CRD

Hospitalid	Beds_category	Teaching	Region	Patients	Type
122	>= 500	0	South	175	Training
176		0		80	Training
382	>= 500	0		213	Training
413		0	West	223	Training
416	>= 500	1	Midwest	231	Training
420	>= 500	1	Northeast	148	Training
73	>= 500	1	Midwest	450	Validation
110	100 - 249	0	South	45	Validation
142	>= 500	0	South	102	Validation
148	250 - 499	0	West	167	Validation
152	100 - 249	0	West	188	Validation
165	>= 500	0	West	71	Validation
167	>= 500	1	West	152	Validation
183	>= 500	0	South	47	Validation
188	>= 500	1	South	184	Validation
195	250 - 499	1	South	212	Validation
197		0	South	139	Validation
198	>= 500	1	South	142	Validation
208	>= 500	0	South	97	Validation
212		1		3	Validation
217	250 - 499	0	South	7	Validation
227	250 - 499	0	West	127	Validation
248	100 - 249	0	Midwest	90	Validation
252	>= 500	1	Midwest	130	Validation
254		0	Midwest	21	Validation
264	>= 500	1	Midwest	3	Validation
281	250 - 499	0	Midwest	110	Validation
282	100 - 249	0	Midwest	40	Validation
300	>= 500	1	Midwest	106	Validation
318	250 - 499	0	South	20	Validation
336	100 - 249	0	Midwest	6	Validation
365		1	Midwest	1	Validation
443	>= 500	1	South	65	Validation
458	>= 500	0	South	213	Validation

Table S2 All included features

Category	Feature name
Basic information	Age, Admission type, BMI, Ethnicity, Height, Gender, pre-ICU admission day, Weight
Treatment received	CABG surgery, Valve surgery, CABG and Valve surgery, Vasopressor, PEEP (max), Plateau pressure (max), Tidal volume (max)
Laboratory test	Albumin (min), Anion gap (max), Base excess (min), Bicarbonate (min), Bilirubin (max), BNP (max), BUN (max), Chloride (min), Creatinine (max), Glucose (avg), Hematocrit (max), Hemoglobin (min), INR (min), Lactate (max), Lymphocytes (avg), Magnesium (max), Neutrophils (min), $PaCO_2$ (max), PaO_2 (min), PaO_2/FiO_2 ratio (min), PH (min), Platelet (min), Potassium (max), PT (max), PTT (max), Sodium (max), Troponin (max), WBC (max)
Vital sign	CVP (avg), FiO2 (max), GCS (min), Heart rate (avg), MBP (avg), Respiratory rate (avg), SaO_2 (min), SBP (avg), SpO_2 (min), Temperature (avg)
Output	Urine output (sum)
Clinical score	Charlson comorbidity index, VAS (max), SOFA (max)

CABG, Coronary artery bypass grafting; PEEP, Positive end expiratory pressure; BNP, Brain natriuretic peptide; BUN, Blood urea nitrogen; PaO2, Partial oxygen pressure in artery; PaCO2, Partial pressure of carbon dioxide in artery; FiO2, Fraction of inspiration O2; PT, Prothrombin time; PTT, Partial thromboplastin time; WBC, White blood cell; CVP, Central venous pressure; GCS, Glasgow Coma Scale; MBP, Mean blood pressure; SaO2, Oxygen saturation in artery; SBP, Systolic blood pressure; SPO2, Pulse oxygen saturation; VAS, Visual Analog Scale; SOFA, Sequential Organ Failure Assessment.

Table S3 Missing ratio of three cohorts

**Basic information**			
Age	0	0	0
Admission type	0	0	0
BMI	2.6	3.1	3.4
Gender	0	0	0
Pre-ICU admission day	0	0	0
Height	2.5	3.1	0.3
Weight	0.3	0.2	3.1
**Treatment received**			
CABG surgery	0	0	0
CABG and Valve surgery	0	0	0
PEEP (max)	9.3	3.4	38.5
Plateau pressure (max)	20.8	12.8	76.3
Valve surgery	0	0	0
Vasopressor	0	0	0
Tidal volume (max)	11.9	4.3	48.3
**Laboratory test**			
Anion gap (max)	38.9	36.9	46.2

Base excess (min)	20.5	15	29.1
Bicarbonate (min)	19.8	12.1	36.8
BUN (max)	16.7	10.4	36.7
Chloride (min)	18.7	12.1	35.1
Creatinine (max)	15.8	10.4	36.6
Hematocrit (max)	5.7	5.7	12.1
Hemoglobin (min)	14.6	13.5	11.5
Lactate (max)	36.9	27.5	84.3
PaCO2 (max)	2.7	1.9	11.2
PaO2 (min)	3.2	2.2	13
PaO2/FiO2 ratio	5.9	5.5	20.8
PH (min)	2.8	1.9	11.2
Platelet (min)	7.8	4.7	19.2
Potassium (max)	30.8	35.3	6.6
WBC (max)	15.5	14	22.3
**Vital sign**			
Heart rate (avg)	2.8	0.4	8.2
CVP (avg)	10.7	4.3	28
FiO2 (max)	0	0	0
GCS (min)	10.4	1.4	37.3
MBP (avg)	0.3	0.4	0.2
Respiratory rate (avg)	6.2	0.4	10.4
SaO2 (min)	29.8	28.1	28.9
SBP (avg)	0.5	0.6	0.2
SpO2 (min)	2.9	0.5	9.3
Temperature (avg)	15	15.2	1.9
**Output**			
Urine output (sum)	9.2	1.6	49.3
**Clinical score**			
Charlson comorbidity index	0	0	0
SOFA (max)	0	0	0
VAS (max)	58.2	56.3	68.8

CABG, Coronary artery bypass grafting; PEEP, Positive end expiratory pressure; BUN, Blood urea nitrogen; PaO2, Partial oxygen pressure in artery; PaCO2, Partial pressure of carbon dioxide in artery; FiO2, Fraction of inspiration O2; WBC, White blood cell; CVP, Central venous pressure; GCS, Glasgow Coma Scale; MBP, Mean blood pressure; PT, Prothrombin time; PTT, Partial thromboplastin time; WBC, White blood cell; CVP, Central venous pressure; GCS, Glasgow Coma Scale; MBP, Mean blood pressure; SaO2, Oxygen saturation in artery; SBP, Systolic blood pressure; SPO2, Pulse oxygen saturation; SOFA, Sequential Organ Failure Assessment; VAS, Visual Analog Scale;

Table S4 The characteristic of the training set

	Non-PI (5732, 88.2%)	PI (766, 11.8%)	P-Value
Age (year)	68.0 [60.0,76.0]	71.0 [62.2,78.0]	<0.001
Female, n (%)	1645 (28.7)	290 (37.9)	<0.001
BMI (kg/m^2)	28.6 [25.3,32.4]	29.3 [25.3,34.0]	0.007
Unplanned admission, n (%)	4072 (71.0)	579 (75.6)	0.010
Ethnicity, n (%)			
Asian	128 (2.2)	19 (2.5)	0.354
Black	202 (3.5)	32 (4.2)	
Hispanic	127 (2.2)	21 (2.7)	
Other	1022 (17.8)	152 (19.8)	

White	4253 (74.2)	542 (70.8)	
CABG, n (%)	4045 (70.6)	543 (70.9)	0.889
Valve surgery, n (%)	2510 (43.8)	418 (54.6)	<0.001
CABG & Valve, n (%)	823 (14.4)	195 (25.5)	<0.001
Vasopressor, n (%)	1205 (21.0)	404 (52.7)	<0.001
PEEP (cmH_2O)	5.0 [5.0,5.0]	5.0 [5.0,10.0]	<0.001
Plateau pressure (cmH_2O)	18.0 [12.0,21.0]	22.5 [20.0,26.0]	<0.001
Tidal volume (mL)	550.0 [400.0,650.0]	600.0 [516.0,697.2]	<0.001
Invasive MV (hour)	5.3 [0.9,11.0]	48.8 [32.1,114.8]	<0.001
Invasive MV >48hours, n (%)		390 (50.9)	<0.001
CCI score	4.0 [3.0,6.0]	5.0 [4.0,7.0]	<0.001
VAS	5.0 [3.0,7.0]	3.0 [1.0,5.0]	<0.001
SOFA score	6.0 [4.0,8.0]	8.0 [6.0,11.0]	<0.001
Pre-ICU admission (day)	0.7 [0.2,2.7]	0.7 [0.1,2.6]	0.047
ICU duration (day)	2.0 [1.2,3.0]	6.3 [3.9,11.7]	<0.001
Hospital duration (day)	6.9 [5.3,9.6]	12.9 [8.6,20.7]	<0.001

CABG, Coronary artery bypass grafting; PEEP, positive end expiratory pressure; MV, mechanical ventilation; CCI, Charlson Comorbidity Index; VAS, Visual Analog Scale; SOFA, Sequential Organ Failure Assessment; ICU, intensive care unit

Table S5 The characteristic of the test set

	Non-PI (4772, 87.9%)	PI (657, 12.1%)	P-Value
Age (year)	68.0 [60.0,75.0]	72.0 [62.0,79.0]	<0.001
Female, n (%)	1343 (28.1)	240 (36.5)	<0.001
BMI (kg/m^2)	28.6 [25.4,32.5]	29.1 [25.5,33.7]	0.008
Unplanned admission, n (%)	3376 (70.7)	498 (75.8)	0.008
Ethnicity, n (%)			
Asian	104 (2.2)	10 (1.5)	0.009
Black	152 (3.2)	30 (4.6)	
Hispanic	116 (2.4)	17 (2.6)	
Other	969 (20.3)	165 (25.1)	
White	3431 (71.9)	435 (66.2)	
CABG, n (%)	3463 (72.6)	438 (66.7)	0.002
Valve surgery, n (%)	1953 (40.9)	381 (58.0)	<0.001
CABG & Valve, n (%)	644 (13.5)	162 (24.7)	<0.001
Vasopressor, n (%)	1104 (23.1)	394 (60.0)	<0.001
PEEP (cmH_2O)	5.0 [0.0,5.0]	5.0 [5.0,10.0]	<0.001
Plateau pressure (cmH_2O)	17.2 [0.0,21.0]	23.0 [19.0,26.0]	<0.001
Tidal volume (mL)	548.0 [0.0,650.0]	588.0 [506.0,675.0]	<0.001
Invasive MV (hour)	5.0 [0.0,10.8]	58.0 [33.7,138.5]	<0.001
Invasive MV >48hours, n (%)		370 (56.3)	<0.001
CCI score	5.0 [3.0,6.0]	6.0 [4.0,7.0]	<0.001
VAS	5.0 [3.0,7.0]	3.0 [1.0,5.0]	<0.001
SOFA score	6.0 [4.0,8.0]	9.0 [6.0,11.0]	<0.001
Pre-ICU admission (day)	0.8 [0.1,2.7]	0.8 [0.1,2.9]	0.207
ICU duration (day)	1.9 [1.2,3.0]	7.2 [4.2,15.1]	<0.001

Hospital duration (day)	7.0 [5.3,9.8]	14.6 [9.6,22.5]	<0.001

CABG, Coronary artery bypass grafting; PEEP, positive end expiratory pressure; MV, mechanical ventilation; CCI, Charlson Comorbidity Index; VAS, Visual Analog Scale; SOFA, Sequential Organ Failure Assessment; ICU, intensive care unit

Table S6 The characteristic of the external validation set

	Non-PI (2423, 82.5%)	PI (515, 17.5%)	P-Value
Age (year)	68.0 [60.0,77.0]	68.0 [61.0,75.5]	0.297
Female, n (%)	801 (33.1)	204 (39.6)	0.005
BMI (kg/m^2)	28.4 [24.7,33.0]	29.7 [25.4,35.4]	<0.001
Unplanned admission, n (%)	1889 (78.0)	369 (71.7)	0.002
Ethnicity, n (%)			
Asian	29 (1.2)	8 (1.6)	<0.001
Black	169 (7.0)	77 (15.0)	
Hispanic	87 (3.6)	16 (3.1)	
Other	125 (5.2)	39 (7.6)	
White	2013 (83.1)	375 (72.8)	
CABG, n (%)	1536 (63.4)	330 (64.1)	0.808
Valve surgery, n (%)	1158 (47.8)	274 (53.2)	0.029
CABG & Valve, n (%)	271 (11.2)	89 (17.3)	<0.001
Vasopressor, n (%)	345 (14.2)	68 (13.2)	0.587
PEEP (cmH$_2$O)	5.0 [5.0,5.0]	5.0 [5.0,8.0]	<0.001
Plateau pressure (cmH$_2$O)	20.0 [16.0,22.0]	22.0 [19.0,25.0]	<0.001
Tidal volume (mL)	564.0 [485.0,658.0]	600.0 [500.0,700.0]	<0.001
Invasive MV (hour)	6.0 [2.8,11.5]	46.0 [29.6,81.9]	<0.001
Invasive MV >48hours, n (%)		246 (47.8)	<0.001
CCI score	3.0 [2.0,4.0]	4.0 [3.0,4.0]	<0.001
VAS	4.0 [1.0,5.0]	3.0 [0.5,5.0]	0.024
SOFA score	7.0 [5.0,9.0]	8.0 [7.0,10.0]	<0.001
Pre-ICU admission (day)	0.4 [0.2,2.1]	0.9 [0.3,3.2]	<0.001
ICU duration (day)	2.0 [1.3,2.9]	4.7 [2.9,7.8]	<0.001
Hospital duration (day)	6.7 [5.1,9.4]	10.9 [7.4,16.6]	<0.001

CABG, Coronary artery bypass grafting; PEEP, positive end expiratory pressure; MV, mechanical ventilation; CCI, Charlson Comorbidity Index; VAS, Visual Analog Scale; SOFA, Sequential Organ Failure Assessment; ICU, intensive care unit

Table S7 The calculation of variance inflation factor

Variable names	VIF
Plateau pressure	3.098629548
Lactate	1.477643031
CCI score	1.191929767
SOFA	1.441165723
CVP	1.193515928
Anion gap	1.206871959
PEEP	2.803971116
Vasopressor	1.439881829
VAS	1.189884918
PH (>7.3)	1.138513732
BUN	1.22219503

CCI, Charlson Comorbidity Index; SOFA, Sequential Organ Failure Assessment; CVP, central venous pressure;

PEEP, positive end expiratory pressure; VAS, Visual Analog Scale; BUN, blood urea nitrogen

Table S8 Performance metrics

Metric (95% CI)	Training set	Test set	External validation set
AUROC	0.853 [0.840, 0.865]	0.867 [0.853, 0.882]	0.704 [0.679, 0.727]
Sensitivity	0.824 [0.796, 0.850]	0.822 [0.793, 0.851]	0.652 [0.612, 0.691]
Specificity	0.700 [0.688, 0.712]	0.755 [0.742, 0.767]	0.616 [0.598, 0.636]
AUPRC	0.490 [0.452, 0.528]	0.521 [0.481, 0.561]	0.370 [0.327, 0.414]

AUROC, area under the receiver operating characteristic; AUPRC, area under the precision recall curve

1.2 Supplementary Figures

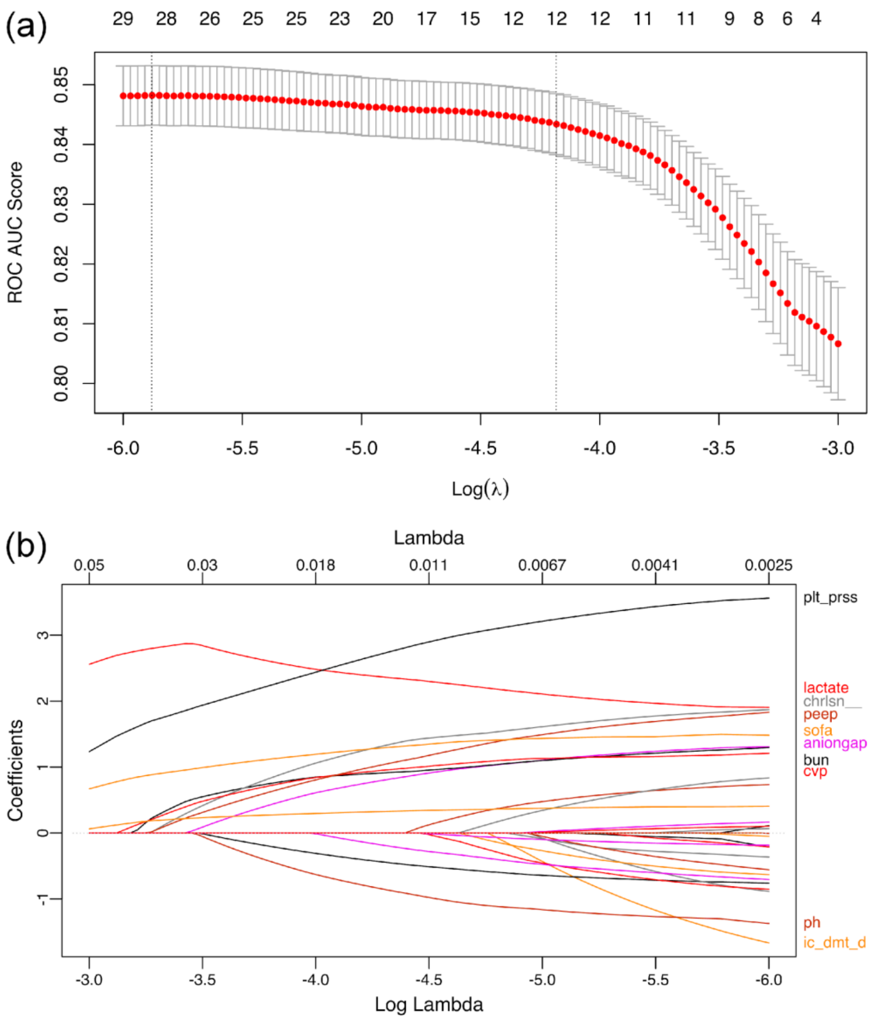

Supplementary Figure 1. Variable selection for PI using the Lasso regression in the training set. (a) using 'lambda.1se' criteria to tune parameters, (b) coefficients of selected variables with lambda parameters.

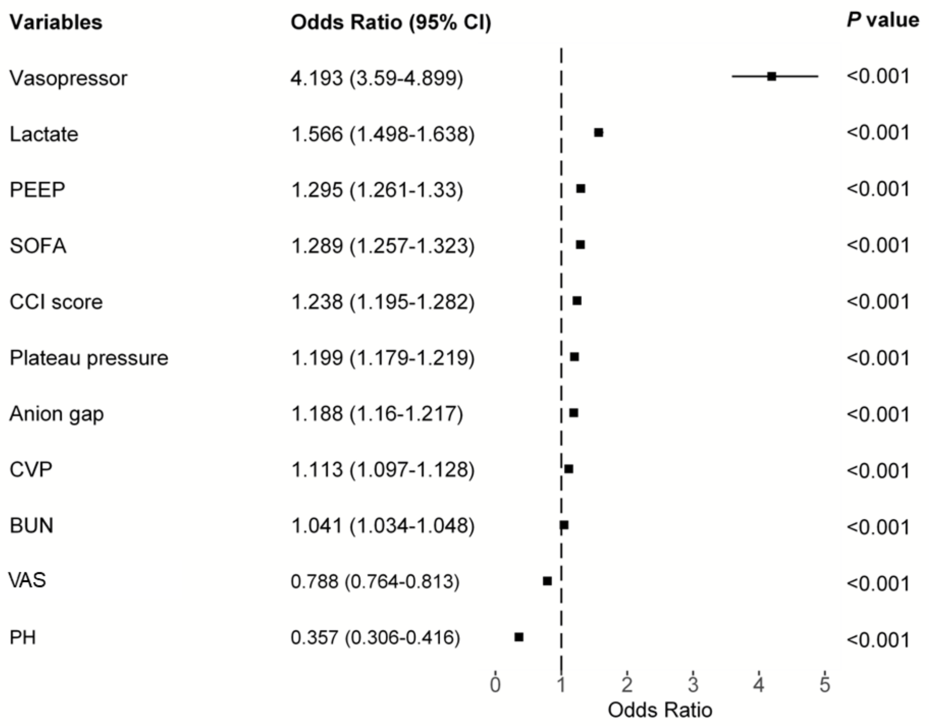

Supplementary Figure 2. Univariable analysis. PEEP, positive end expiratory pressure; SOFA, Sequential Organ Failure Assessment; CCI, Charlson Comorbidity Index; CVP, central venous pressure; BUN, blood urea nitrogen; VAS, Visual Analog Scale

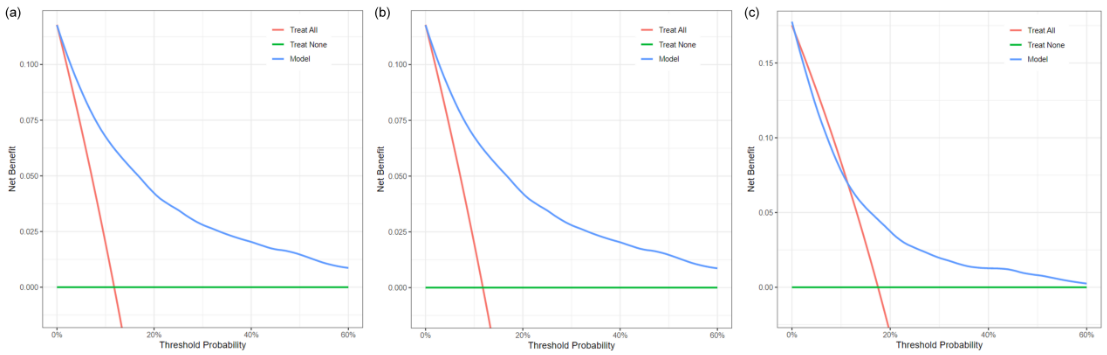

Supplementary Figure 3. Decision curve analysis of the prediction model in study cohorts
